# RNA-seq and miRNA-seq data from pharmacological inhibition of the G9a/GLP histone methyltransferase complex with UNC0642 in SAMP8 mice

**DOI:** 10.1016/j.dib.2021.107114

**Published:** 2021-05-02

**Authors:** Shrikant Pawar, Aina Bellver-Sanchis, Christian Griñán-Ferré

**Affiliations:** a Department of Genetics, Yale University, New Haven, CT, USA; b Department of Pharmacology and Therapeutic Chemistry, Institut de Neurociències-Universitat de Barcelona, Avda. Joan XXIII, 27, Barcelona 08028, Spain

**Keywords:** Epigenetics, Alzheimer's disease, RNA seq, SAMP8, SAMR1, UNC0642, G9a/GLP

## Abstract

Growing evidence demonstrates the epigenetic modulation as a key event in Alzheimer's disease (AD) pathology. Furthermore, recent data suggests that the epigenetic regulation by the methyltransferase G9a is a crucial mechanism involved in learning and memory formation. Taking this into account, we hereby provide genomics data from pharmacological intervention with UNC0642, a potent and selective G9a/GLP in SAMP8 mice, a model of Alzheimer's disease (AD). We have generated novel RNA-seq and miRNA-seq data for three groups, healthy SAMR1, SAMP8 control and SAMP8 treated with UNC0642 (5 mg/Kg). Thus, the new data can be used to find miRNA regulation, and the mRNA's modified in AD under G9a/GLP inhibition.

## Specifications Table

**Table:** 

Subject	Bioinformatics
Specific subject area	Comparative genomics
Type of data	RNA-seq and miRNA-seq Table and Figures
How data were acquired	RNA-seq and miRNA-seq was generated for three groups, healthy SAMR1, SAMP8 control and SAMP8 treated with UNC0642 (5 mg/Kg)
Data format	Raw & analyzed
Parameters for data collection	Treatment with UNC0642 (5 mg/Kg)
Description of data collection	The experimental features are compared between treatments healthy SAMR1, SAMP8 control and SAMP8 treated with UNC0642 (5 mg/Kg)
Data source location	Department of Pharmacology and Therapeutic Chemistry, Institut de Neurociències-Universitat de Barcelona, Avda. Joan XXIII, 27. 08,028 Barcelona, Spain.
Data accessibility	https://data.mendeley.com/datasets/f47bm47bcw/3 Pawar, Shrikant (2020), “RNA-seq and miRNA-seq data from pharmacological inhibition of the G9a/GLP histone methyltransferase complex with UNC0642”, Mendeley Data, v3, http://dx.doi.org/10.17632/f47bm47bcw.3

## Value of the Data

• This data can be used to find miRNA regulation, and the mRNA's modified in Alzheimer's disease under G9a/GLP inhibition.
• Many researcher groups focusing on how epigenetic modifications contribute to specific aspects of Alzheimer's disease can be benefited with this data.
• Presented genomics data from pharmacological intervention with UNC0642, a potent and selective G9a/GLP in SAMP8 mice, a model of Alzheimer's disease can provide insights into promising pharmacological targets.

## 1 Data Description

We have presented novel RNA-seq and miRNA-seq data generated for three groups, healthy SAMR1, SAMP8 control and SAMP8 treated with UNC0642 (5 mg/Kg) during 4 weeks in drinking water administration. Senescence-accelerated mouse prone 8 (SAMP8) is a naturally occurring mouse line that displays a phenotype of accelerated aging, while SAMR1 mice represent a normal aging control [2], [3], [4]. This new data can be used to find miRNA regulation, and the mRNA's modified in AD under G9a/GLP inhibition. We have provided the overall distribution of significant genes in comparison SAMR1 Ct vs SAMP8 Ct, and comparison SAMP8 Ct vs SAMP8 UNC0642 (5 mg/Kg)), with significant miRNA's (fold change) in a heatmap. The isolation of volume and concentrations for RNA-seq and miRNA-seq experiments is provided in Table 1 and miRNA-seq comparisons are provided in Supplementary files 1, 2, 3 and 4.

**Table 1:** RNA isolation volume and concentrations for RNA-seq and miRNA-seq experiments.

Sample Name	Volume (ul)	Sample Type	Nanodrop (ng/ul)	A260	A280	260/280	RIN
SAMR1 Ct	15	RNA	433.7	10.84	5.286	2.05	5.6
SAMP8 Ct	15	RNA	123	3.076	1.513	2.03	4.6
SAMP8 UNC0642 (5 mg/Kg)	15	RNA	146.9	3.673	1.795	2.05	8.1

### 1.1 RNA-seq data

**Fig. 1:**
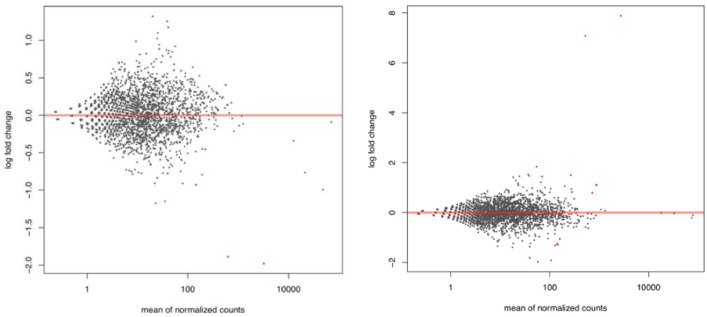
Overall distribution of significant genes (Left: Comparison SAMR1 Ct vs SAMP8 Ct, Right: Comparison SAMP8 Ct vs SAMP8 UNC0642 (5 mg/Kg)).

With comparison, SAMR1 Ct vs SAMP8 Ct, 3600 unique ensemble ids were generated from DESeq2 package (Supplementary file 1). With a fold change of ≧ 0.5, 78 genes were found to be significantly upregulated while 62 genes were found to be downregulated in SAMP8 Ct with a fold change of ≦ −0.5. Fig. 1 (Left) provides overall distribution of these significant genes. With comparison, SAMP8 Ct vs SAMP8 UNC0642, 3126 unique ensemble ids were generated from DESeq2 package (Supplementary file 2). With a fold change of ≧ 0.5, 140 genes were found to be significantly upregulated while 159 genes were found to be downregulated in SAMP8 UNC0642 with a fold change of ≦ −0.5. Fig. 1 (Right) provides overall distribution of these significant genes.

POU class 3 homeobox 2 (POU3F2), SATB homeobox 2 (SATB2), T-box, brain 1 (TBR1), ankyrin repeat domain 52 (ANKRD52), calcium/calmodulin dependent protein kinase II inhibitor 1 (CAMK2N1) where some of the top up regulated genes in SAMP8 treated with UNC0642 (5 mg/Kg), while *T*-cell lymphoma invasion and metastasis 1 (TIAM1), cell adhesion molecule 1 (CADM1), clathrin light chain A (CLTA), importin 7 (IPO7) where some of the top down regulated genes in SAMP8 UNC0642 condition.

### 1.2 miRNA-seq data

**Fig. 2:**
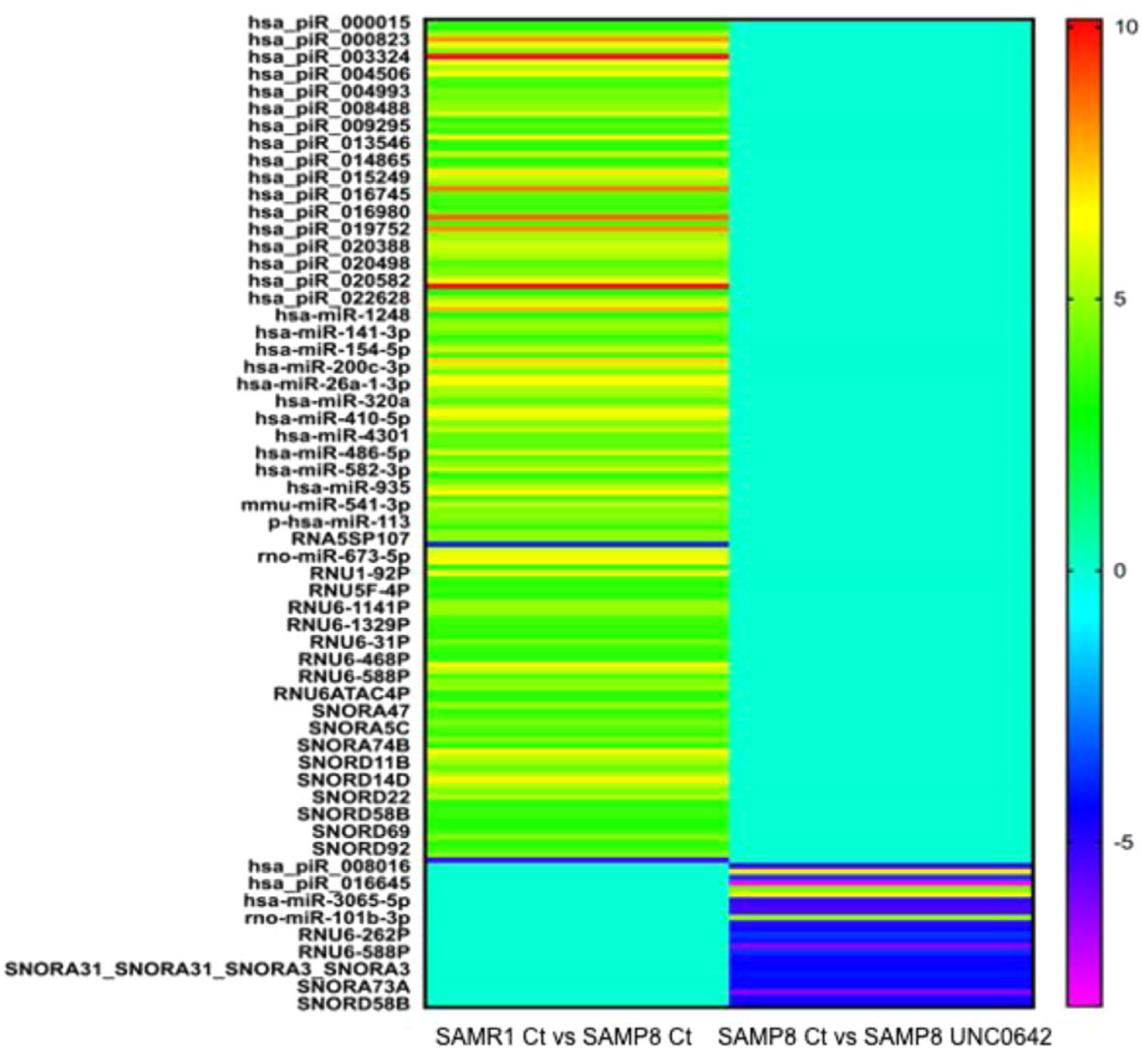
Heatmap (fold change) of significant miRNA's observed in comparisons SAMR1 Ct vs SAMP8 Ct and SAMP8 Ct vs SAMP8 UNC0642 (5 mg/Kg).

Comparison SAMR1 Ct vs SAMP8 Ct had 147 miRNA's, while comparison SAMP8 Ct vs SAMP8 UNC0642 (5 mg/Kg)with 25 miRNA's were found to have a significant P value (<0.05) and fold change (>2). The list of these miRNA's is provided in files Supplementary file 3 and Supplementary file 4. Fig. 2 depicts the heatmap of significant miRNA's observed in compared conditions.

## 2 Data

The early cognitive impairment in Alzheimer's Disease (AD) can be mistaken for premature aging correlating with epigenetic changes occurring at a younger age. Exactly how epigenetic modifications contribute to specific aspects of AD is the focus of many researcher groups. The G9a/GLP complex plays a role in learning and memory since its inhibition participates in the maintenance of long-term potentiation (LTP) and Long-Term Depression (LTD). The inhibition of this complex increases the expression of memory permissive genes such as *Bdnf*, previously repressed, confirming the transcriptional modifications modulated by G9a/GLP complex. Due to the transcription repression produced by G9a/GLP through its repressive mark H3K9me2 and given the evidence demonstrating the implication of the G9a/GLP complex in different human diseases, it has emerged as a promising pharmacological target [1]. Therefore, chromatin modifications induced by drugs could offer an opportunity for novel therapeutic strategies in AD.

## 3 Experimental Design, Materials and Methods

### 3.1 Design

Total RNA from cells was isolated with Quick Biology's miRNA-seq [5]. One of the biggest limitations to the small RNA library protocol are the insert-less 5′adapter and 3′adapter ligations that form what are called adapter dimers. These dimers compete for resources during RT-PCR. When over-amplified, these ~120 bp products can contaminate true 150 bp microRNA library bands. Quick Biology's optimized protocol utilizing modified oligonucleotides is used to eliminate the reverse-transcription of adapter dimers. We used multiplex miRNA libraries in one run of Illumina sequencing, to determine the level of multiplexing or number of libraries to pool together, yielding reads between 5 and 10 million reads per library. Sequence small RNA libraries using a 1 × 50 or 1 × 75 bp sequencing run were utilized, and for miRNA expression profiling, 100K–5 M mapped reads per sample was considered.

### 3.2 RNA-seq data

We have separated control and treatment groups into 3 categories, SAMR1 Ct are a healthy mice control group, SAMP8 Ct are control mice group, and SAMP8 UNC0642 (5 mg/Kg) are treated mice group. RNA isolation volume and concentrations are provided in Table 1. Two replicates for samples SAMR1 Ct, SAMP8 Ct and SAMP8 UNC0642 (5 mg/Kg) were aligned with reference genome using HISAT aligner. It is a fast and sensitive spliced alignment program based on the Burrows-Wheeler transform and hierarchical indexing employing one global FM index representing the whole genome, and many separate local FM indexes for small regions collectively covering the genome. All the aligned bam files were separately processed through FeatureCounts for calculating counts for each identifier. FeatureCounts is a lightweight read counting program that can be used to count both gDNA-seq and RNA-seq reads for genomic features in SAM/BAM files. Finally, DESeq2 package was implemented to test for differential expression between conditions, SAMR1 Ct vs SAMP8 Ct and SAMP8 Ct vs SAMP8 UNC0642 (5 mg/Kg). DESeq2 estimates variance-mean dependence in count data from high-throughput sequencing assays and test for differential expression based on a model using the negative binomial distribution.

### 3.3 miRNA-seq data

miRNA was analyzed using sRNA Detection module of Oasis [6]. It examines sample qualities, as well as quantifies known and novel sRNAs for each submitted sample. A principal component analysis (PCA) plot was generated to understand sample similarities based on sRNA expression, followed by trimming reads filtered for being too short or too long. sRNAs in Oasis are grouped into various species, including micro RNA (miRNA), piwi RNA (piRNA), small nucleolar RNA (snoRNA), small nuclear RNA (snRNA) and ribosomal RNA (rRNA). Trimming is followed by length filtering and mapping reads to the reference genome. Finally count files for all known sRNA species, as well as novel, predicted miRNAs, are generated for each sample. Differential miRNA analysis was performed using package DESeq(dds), followed by sizeFactors(dds) and dispersions(dds) [7], [8].

## 4 Transparency Document

Supplementary file 1, 2, 3, 4.

## Ethics Statement

All experiments comply with the ARRIVE guidelines and carried out in accordance with the U.K. Animals (Scientific Procedures) Act, 1986 and associated guidelines, EU Directive 2010/63/EU for animal experiments, or the National Institutes of Health guide for the care and use of Laboratory animals (NIH Publications No. 8023, revised 1978). Female mice were utilized for this study for all the three groups, control healthy SAMR1, SAMP8 control and SAMP8 treated with UNC0642.

## Declaration of Competing Interest

The authors declare that they have no known competing financial interests or personal relationships which have or could be perceived to have influenced the work reported in this article.

## Appendix Supplementary Materials

Image, application 1

## Footnotes

Supplementary material associated with this article can be found in the online version at doi:10.1016/j.dib.2021.107114.
